# Misconceptions of the *p*-value among Chilean and Italian Academic Psychologists

**DOI:** 10.3389/fpsyg.2016.01247

**Published:** 2016-08-23

**Authors:** Laura Badenes-Ribera, Dolores Frias-Navarro, Bryan Iotti, Amparo Bonilla-Campos, Claudio Longobardi

**Affiliations:** ^1^Department of Methodology of the Behavioral Sciences, University of ValenciaValencia, Spain; ^2^Veterinary and Prevention Department, University of TurinTurin, Italy; ^3^Department of Psychology, University of TurinTurin, Italy; ^4^Research Center on Development and Educational, Faculdade Européia de VitòriaCariacica, Brazil

**Keywords:** *p*-value misconceptions, survey, statistical cognition, education, high education

## Abstract

Common misconceptions of *p*-values are based on certain beliefs and attributions about the significance of the results. Thus, they affect the professionals' decisions and jeopardize the quality of interventions and the accumulation of valid scientific knowledge. We conducted a survey on 164 academic psychologists (134 Italian, 30 Chilean) questioned on this topic. Our findings are consistent with previous research and suggest that some participants do not know how to correctly interpret *p*-values. The inverse probability fallacy presents the greatest comprehension problems, followed by the replication fallacy. These results highlight the importance of the statistical re-education of researchers. Recommendations for improving statistical cognition are proposed.

## Introduction

Null hypothesis significance testing (NHST) is one of the most widely used methods for testing hypotheses in psychological research (Cumming et al., 2007). NHST is a hybrid of two highly influential but incompatible schools of thought in modern statistics: tests of significance, developed by Fisher, and tests of statistical hypotheses, developed by Neyman and Pearson (Gigerenzer and Murray, 1987; Perezgonzalez, 2015). The *p*-value linked to the results of a statistical test is the probability of witnessing the observed result or a more extreme value if the null hypothesis is true (Hubbard and Lindsay, 2008; Kline, 2013). The definition is clear and precise. Nevertheless, previous studies indicate that academic psychologists often do not interpret *p*-values correctly (Oakes, 1986; Haller and Krauss, 2002; Badenes-Ribera et al., 2015a).

The misconceptions of *p*-values are made based on certain beliefs and attributions about the significance of the results. These beliefs and attributions could influence the methodological behavior of the researcher, affect the decisions of the professional and jeopardize the quality of interventions and the accumulation of valid scientific knowledge. Therefore, interpretation of the findings should not be susceptible to erroneous beliefs.

The most common misconceptions about *p*-value are “inverse probability fallacy,” “replication fallacy,” “effect size fallacy,” and “clinical or practical significance fallacy” (Carver, 1978; Cohen, 1994; Kirk, 1996; Nickerson, 2000; Kline, 2013).

The “inverse probability fallacy” is the false belief that the *p*-value indicates the probability that the null hypothesis (H_0_) is true, given certain data (Pr(H_0_| Data)). Essentially, it means confusing the probability of the result, assuming that the null hypothesis is true, with the probability of the null hypothesis, given certain data.

The “replication fallacy” is the belief that the *p*-value is the degree of replicability of the result and its complement, 1-*p*, is often interpreted as an indication of the exact probability of replication (Carver, 1978; Nickerson, 2000). Therefore, a result with a *p*-value of 0.05 would mean that 95 out of 100 times the statistically significant results observed will be maintained in future studies (Fidler, 2005). Nevertheless, “any *p*-value gives only very vague information about what is likely to happen on replication, and any single *p*-value could easily have been quite different, simply because of sampling variability” (Cumming, 2008, p. 286).

The “effect size” fallacy involves the belief that the *p*-value provides direct information about the effect magnitude (Gliner et al., 2001). In this way, the researchers believe that when *p* is smaller, the effect sizes are larger. Instead, the effect size can only be determined by directly estimating its value with the appropriate statistic and its confidence interval (Cohen, 1994; Cumming, 2012; Kline, 2013).

Finally, the “clinical or practical significance” fallacy links statistical significance to the importance of the effect size (Nickerson, 2000). That is, a statistically significant effect is interpreted as an important effect. However, a statistically significant effect can be without clinical importance, and vice versa. Therefore, the clinical or practical importance of the findings should be described by an expert in the field, and not presented by the statistics alone (Kirk, 1996).

The present study analyzes if the most common misconceptions of *p*-values are made by academic psychologists from a different geographic area than the original researches (Spain by Badenes-Ribera et al., 2015a Germany by Haller and Krauss, 2002; United States of America by Oakes, 1986). Knowing the prevalence and category of the misinterpretations of *p*-values is important for deciding and planning statistical education strategies designed to right incorrect interpretations. This is especially important for psychologists in academia, considering that through their teaching activities they will influence many students that will have a professional future in the field of Psychology. Thus, their interpretation of the results should not be derived from erroneous beliefs.

To address this question, we carried out a replication study. Several authors have pointed out that replication is the most objective method for checking if the result of a study is reliable and this concept plays an essential role in the advance of scientific knowledge (Carver, 1978; Wilkinson and The Task Force on Statistical, Inference, 1999; Nickerson, 2000; Hubbard, 2004; Cumming, 2008; Hubbard and Lindsay, 2008; Asendorpt et al., 2013; Kline, 2013; Stroebe and Strack, 2014; Earp and Trafimow, 2015).

The replication includes repetitions by different researchers in different places with incidental or deliberate changes to the experiment (Cumming, 2008). As Stroebe and Strack (2014) state, “exact replications are replications of an experiment that operationalize both the independent and the dependent variable in exactly the same way as the original study” (p. 60). Therefore, in direct replication, the only differences between the original and replication studies would be the participants, location and moment in time. “In contrast, conceptual replications try to operationalize the underlying theoretical variables using different manipulations and/or different measures” (Stroebe and Strack, 2014, p. 60). Like in conceptual replication, “replications with extensions examine the scope and limits of initial findings to see if they are capable of being generalized to other populations, geographic areas, time periods, measurement instruments, and the like” (Hubbard and Ryan, 2000, p. 676). Consequently, conceptual replication or replications with extensions are a better route for assessing the reliability, validity, and generalizability of empirical findings (Hubbard, 2004; Stroebe and Strack, 2014; Earp and Trafimow, 2015).

In this light, our work is a replication of the study by Badenes-Ribera et al. (2015a) which analyzed the diffusion of the most common misconceptions of *p*-value and two correct interpretations among Spanish academic psychologists. We modified two aspects of original research: the answer scale format of the instrument for measuring misconceptions of *p*-values and the geographical areas of the participants (Italian and Chilean academic psychologists).

In the original study, the instrument included a set of 10 questions that analyzed the interpretations of *p*-value. The questions were posed using the following format: “Suppose that a research article indicates a value of *p* = 0.001 in the results section (alpha = 0.05) […]” and the participants had to mark which of the statements were true or false. Therefore, the response scale format was dichotomous. In our study, we changed the response scale format, and the participants could only indicate which answers were true instead of being forced to opt for a true or false option. This response scale format ensures that answers given by the subjects have a higher level of confidence, but on the other hand it does not allow knowing if the items that were not chosen are considered false or “does not know.”

The second modification was the geographic area. The original research was conducted in Spain, while the present study was carried out in Chile and Italy. Previous studies have shown that the fallacies about *p*-values are common among academic psychologists and university students majoring in psychology from several countries (Germany, USA, Spain, Israel). However, nothing is known about the extension of these misinterpretations in Chile (a Latin-American country) and Italy (another country from Europe union). Consequently, research on misconceptions of *p*-values in these countries is useful to improve our current knowledge about the extension of the fallacies among academic psychologists. Furthermore, the present study is part of a cross-cultural research project between Spain and Italy about statistical cognition, and it is framed within the line of research on cognition and statistical education that our research group has been developing for many years.

## Methods

### Participants

A non-probabilistic (convenience) sample was used. The sample initially comprised 194 academic psychologists from Chile and Italy. Of these 194 participants, thirty did not respond to questions about misconceptions of *p*-values and were removed from the analysis. Consequently, the final sample consisted of 164 academic psychologists; 134 of them were Italian and 30 were Chilean. Table 1 presents a description of the participants.

**Table 1:** **Description of the participants**.

	**Chile (*****n*** = 30**)**		**Italy (*****n*** = 134**)**	
	***n***	**%**	***n***	**%**
**SEX**				
Men	15	50	62	46.27
Women	15	50	72	53.73
**PSYCHOLOGY KNOWLEDGE AREAS**				
Development and educational psychology,	7	23.33	25	18.66
Clinical and dynamic psychology	7	23.33	23	17.16
Social psychology	5	16.67	22	16.42
Methodology	5	16.67	13	9.7
Neuropsychology	1	3.33	14	10.45
Work and organizational psychology	3	10	10	7.46
General psychology	2	6.67	27	20.15
**TYPE OF UNIVERSITY**				
Public	13	43.33	116	86.57
Private	17	56.67	18	13.43
**HAVE YOU BEEN REVIEWERS FOR SCIENTIFIC JOURNALS IN THE LAST YEAR?**				
Yes	17	56.67	115	85.82
No	13	43.33	19	14.18

Of the 134 Italians participants, 46.3% were men and 53.7% were women, with a mean age of 48.35 years (*SD* = 10.65). The mean number of years that the professors had spent in academia was 13.28 years (*SD* = 10.52).

Of the 30 Chilean academic psychologists, men represented 50% of the sample. In addition, the mean age of the participants was 44.50 years (*SD* = 9.23). The mean number of years that the professors had spent in academia was 15.53 years (*SD* = 8.69).

### Instrument

We prepared a structured questionnaire that included items related to information about sex, age, years of experience as an academic psychologist, Psychology knowledge area, as well as items related to the methodological behavior of the researcher.

The instrument then included a set of 10 questions that analyze the interpretations of the *p*-value (Badenes-Ribera et al., 2015a). The questions are posed using the following argument format: “Suppose that a research article indicates a value of *p* = 0.001 in the results section (α = 0.05). Select true statements.”

A.-Inverse probability fallacy:

1. The null hypothesis has been shown to be true.
2. The null hypothesis has been shown to be false.
3. The probability of the null hypothesis has been determined (*p* < 0.001).
4. The probability of the experimental hypothesis has been deduced (*p* < 0.001).
5. The probability that the null hypothesis is true, given the data obtained, is 0.01.

B.-Replication fallacy:

6. A later replication would have a probability of 0.999 (1-0.001) of being significant.

C.-Effect size fallacy:

7. The value *p* < 0.001 directly confirms that the effect size was large.

D.-Clinical or practical significance fallacy:

8. Obtaining a statistically significant result indirectly implies that the effect detected is important.

E.-Correct interpretation and decision made:

9. The probability of the result of the statistical test is known, assuming that the null hypothesis is true.
10. Given that *p* = 0.001, the result obtained makes it possible to conclude that the differences are not due to chance.

The questions were administered in Italian and Spanish respectively. Original items were in Spanish, and therefore all the items were translated into Italian by applying the standard back-translation procedure, which implied translations from Spanish to Italian and vice versa (Balluerka et al., 2007).

Finally, the instrument evaluates other questions, such as statistical practice or knowledge about the statistical reform, which are not analyzed in this paper.

### Procedure

The e-mail addresses of academic psychologists were found by consulting the websites of Chilean and Italian universities, resulting in 2321 potential participants (1824 Italians, 497 Chilean). The data collection was performed from March to May 2015. Potential participants were invited to complete a survey through the use of a CAWI (Computer Assisted Web Interviewing) system. A follow-up message was sent 2 weeks later to non-respondents. Individual informed consent was also collected from academics along with written consent describing the nature and objective of the study according to the ethical code of the Italian Association for Psychology (AIP). The consent stated that data confidentiality would be assured and that participation was voluntary. Thirty participants (25 Italian and 5 Chilean) did not respond to questions about *p*-value misconceptions and were therefore removed from the study. The response rate was 7.07% (Italian 7.35%, Chilean 6.04%).

### Data analysis

The analysis included descriptive statistics for the variables under evaluation. To calculate the confidence interval for percentages, we used score methods based on the works of Newcombe (2012). These methods perform better than traditional approaches when calculating the confidence intervals of percentages. These analyses were performed with the statistical program IBM SPSS v. 20 for Windows.

## Results

Table 2 presents the percentage of responses by participants who endorse the eight false statements about the *p*-values, according to the Psychology knowledge areas and nationality of the participants.

**Table 2:** **Percentage of participants who endorsed the false statements (and 95% Confidence Intervals)**.

	**Chile (*****n*** = 30**)**				**Italy (*****n*** = 134**)**				**Total (*****N*** = 164**)**					
**Item**	**Methodology (*n* = 5)**		**Other knowledge (*n* = 25)**		**Methodology (*n* = 13)**		**Other knowledge areas (*n* = 121)**		**Methodology (*n* = 18)**		**Other knowledge areas (*n* = 146)**		**Total (*N* = 164)**	
	***n***	**%**	***n***	**%**	***n***	**%**	***n***	**%**	***n***	**%**	***n***	**%**	***n***	**%**
**INVERSE PROBABILITY FALLACY**														
1. The null hypothesis has been shown to be true	0	0 [0, 43.45]	1	4 [0.71, 19.54]	0	0 [0, 22.81]	5	4.13 [1.78, 9.31]	0	0 [0, 17.59]	6	4.11 [1.90, 8.68]	6	3.66 [1.69, 7.75]
2. The null hypothesis has been shown to be false	2	40 [11.76, 76.93]	15	60 [40.74, 76.60]	3	23.08 [8.18, 50.26]	34	28.10 [20.86, 36.69]	5	27.78 [12.50, 50.87]	49	33.56 [26.41, 42.56]	54	32.93 [26.20, 40.44]
3. The probability of the null hypothesis has been determined (*p* = 0.001)	1	20 [3.62, 62.45]	3	12 [4.17, 29.96]	4	30.77 [12.68, 57.63]	31	25.62 [18.68, 34.06]	5	27.78 [12.50, 50.87]	34	23.29 [17.17, 30.77]	39	23.78 [17.91, 30.85]
4. The probability of the experimental hypothesis has been deduced (*p* = 0.001)	0	0 [0, 43.45]	4	16 [6.40, 34.65]	1	7.69 [1.37, 33.31]	15	12.40 [7.66, 19.45]	1	5.56 [0.99, 25.76]	19	13.01 [8.49, 19.43]	20	12.20 [8.03, 18.09]
5. The probability that the null hypothesis is true, given the data obtained, is 0.01	0	0 [0, 43.45]	2	8 [2.22, 24.97]	3	23.08 [8.18, 50.26]	17	14.05 [8.96, 21.35]	3	16.67 [5.84, 39.22]	19	13.01 [8.49, 19.43]	22	13.41 [9.03, 19.48]
Participants who not endorse the five false statements	3	60 [23.07, 88.24]	7	28 [14.28, 47.58]	5	38.46 [17.71, 64.48]	48	39.67 [31.40, 48.57]	8	44.44 [24.56, 66.28]	55	37.67 [30.22, 45.75]	63	38.41 [31.32, 46.04]
**REPLICATION FALLACY**														
6. A later replication would have a probability of 0.999 (1-0.001) of being significant.	0	0 [0, 43.45]	5	20 [8.86, 39.13]	1	7.69 [1.37, 33.31]	14	11.57 [7.02, 18.49]	1	5.56 [0.99, 25.76]	19	13.01 [8.49, 19.43]	20	12.20 [8.03, 18.09]
**EFFECT SIZE FALLACY**														
7. The value *p* = 0.001 directly confirms that the effect size was large	0	0 [0, 43.45]	0	0 [0, 13.32]	1	7.69 [1.37, 33.31]	7	5.79 [2.83, 11.46]	1	5.56 [0.99, 25.76]	7	4.79 [2.34, 9.57]	8	4.88 [2.49, 9.33]
**CLINICAL OR PRACTICAL FALLACIES**														
8. Obtaining a statistically significant result indirectly implies that the effect detected is important	0	0 [0, 43.45]	2	8 [2.22, 24.97]	1	7.69 [1.37, 33.31]	11	9.09 [5.15, 15.55]	1	5.56 [0.99, 25.76]	13	8.90 [5.28, 14.64]	14	8.54 [5.15, 13.82]

Regarding the “inverse probability fallacy,” the majority of the Italian and Chilean academic psychologists perceived some of the false statements about the *p*-value to be true, like in the study of Badenes-Ribera et al. (2015a).

The participants in the area of Methodology made fewer incorrect interpretations of *p*-values than the rest of the participants. There were, however, overlaps among the confidence intervals; therefore, the differences among the percentage were not statistically significant.

In addition, Italian methodologists presented more problems than their peer in the false statements “The probability of the null hypothesis has been determined *(p* = 0.001) and “The probability that the null hypothesis is true, given the data obtained, is 0.001,” although there were overlaps among the confidence intervals. Consequently, the differences among the percentages were not statistically significant.

Overall, the false statement that received the most support is “The null hypothesis has been shown to be false.” By sample, Italian participants encountered the most problems with the false statement “The probability of the null hypothesis has been determined (*p* = 0.001),” while Chilean participants with “The null hypothesis has been shown to be false.”

Concerning the “replication fallacy,” as Table 2 shows, the majority of the participants (87.80%) correctly evaluated the false statement, like in the studies of Badenes-Ribera et al. (2015a) with 65.3% of correct answers and Haller and Krauss, 2002) with 51% of correct answers. The participants in the area of Methodology had fewer incorrect interpretations of the *p*-value than the rest also in this case, but there were overlaps among the confidence intervals; therefore, the differences among the percentage were not statistically significant.

Regarding the percentage of participant responses that endorsed the false statements about the *p*-value as an effect size and as having clinical or practical significance, it is noteworthy that only a limited percentage of the participants believed that small *p*-values indicates that the result are important and that the *p*-values indicates effect size. These findings are in line with the results of the study of Badenes-Ribera et al. (2015a). By sample, Chilean participants presented fewer misconceptions than Italian participants, however, there were overlaps among the confidence intervals; thus, the differences among the percentages were not statistically significant.

**Figure 1:**
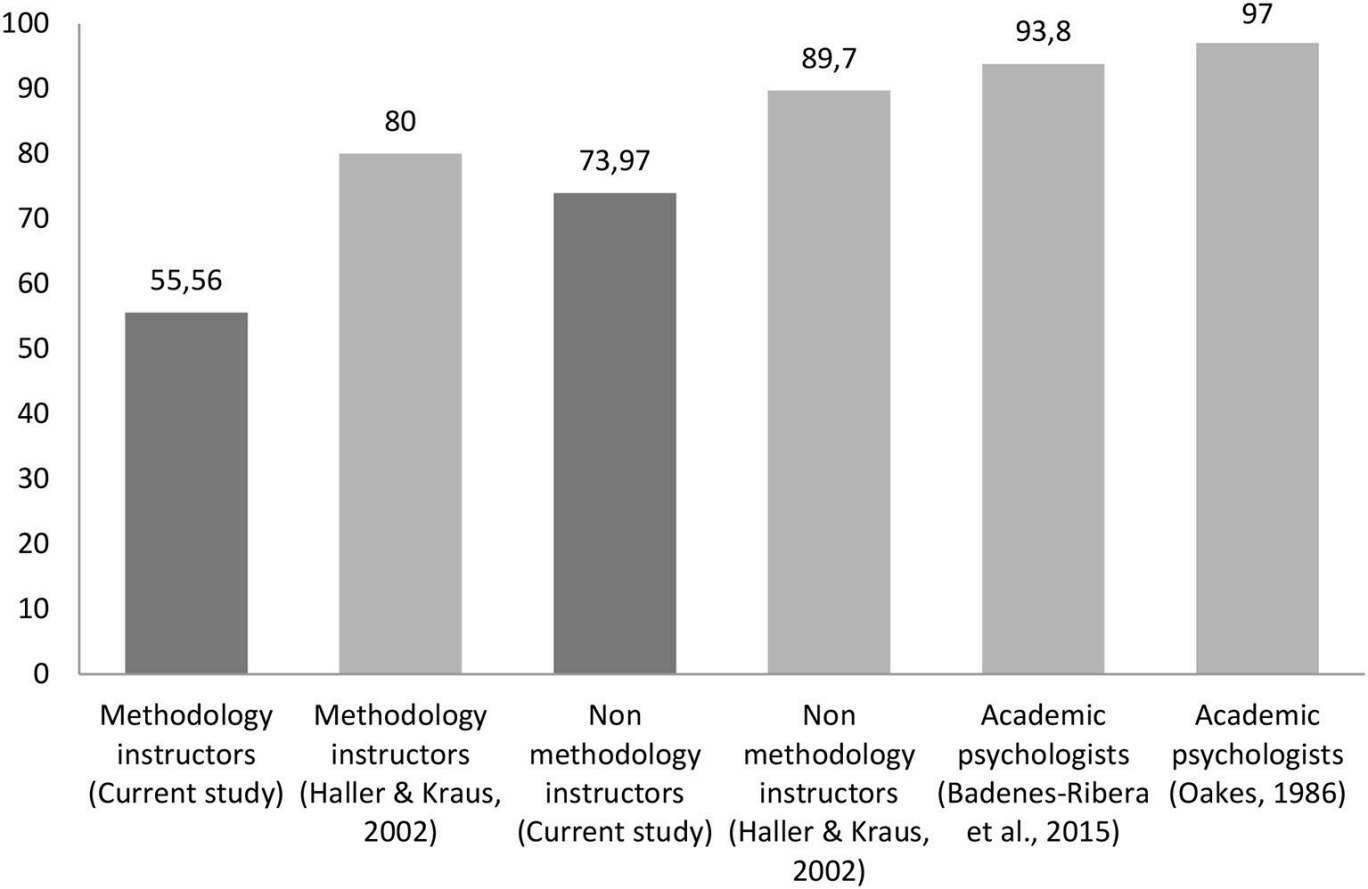
**Percentages of participants in each group who endorse at least one of the false statements in comparison to the studies of Badenes-Ribera et al. (2015a), Haller and Krauss (2002), and Oakes (1986)**.

Figure 1 shows the percentage of participants in each group who endorse some of the false statements in comparison to the studies of Oakes (1986), Haller and Krauss, 2002), and Badenes-Ribera et al. (2015a). The number of statements (and the statements themselves) posed to the participants differed across studies, and this should be borne in mind. The study by Oakes and that of Haller and Kraus presented the same six wrong statements to the participants. In the study of Badenes-Ribera et al. and the current study, the same eight false questions were presented. Overall it is noteworthy that despite the fact that 30 years have passed since the Oakes' original study (1986) and 14 years since the study of Haller and Krauss (2002), and despite publication of numerous articles on the misconceptions of *p*-values, most of the Italian and Chilean academic psychologists do not know how to correctly interpret *p*-values.

**Figure 2:**
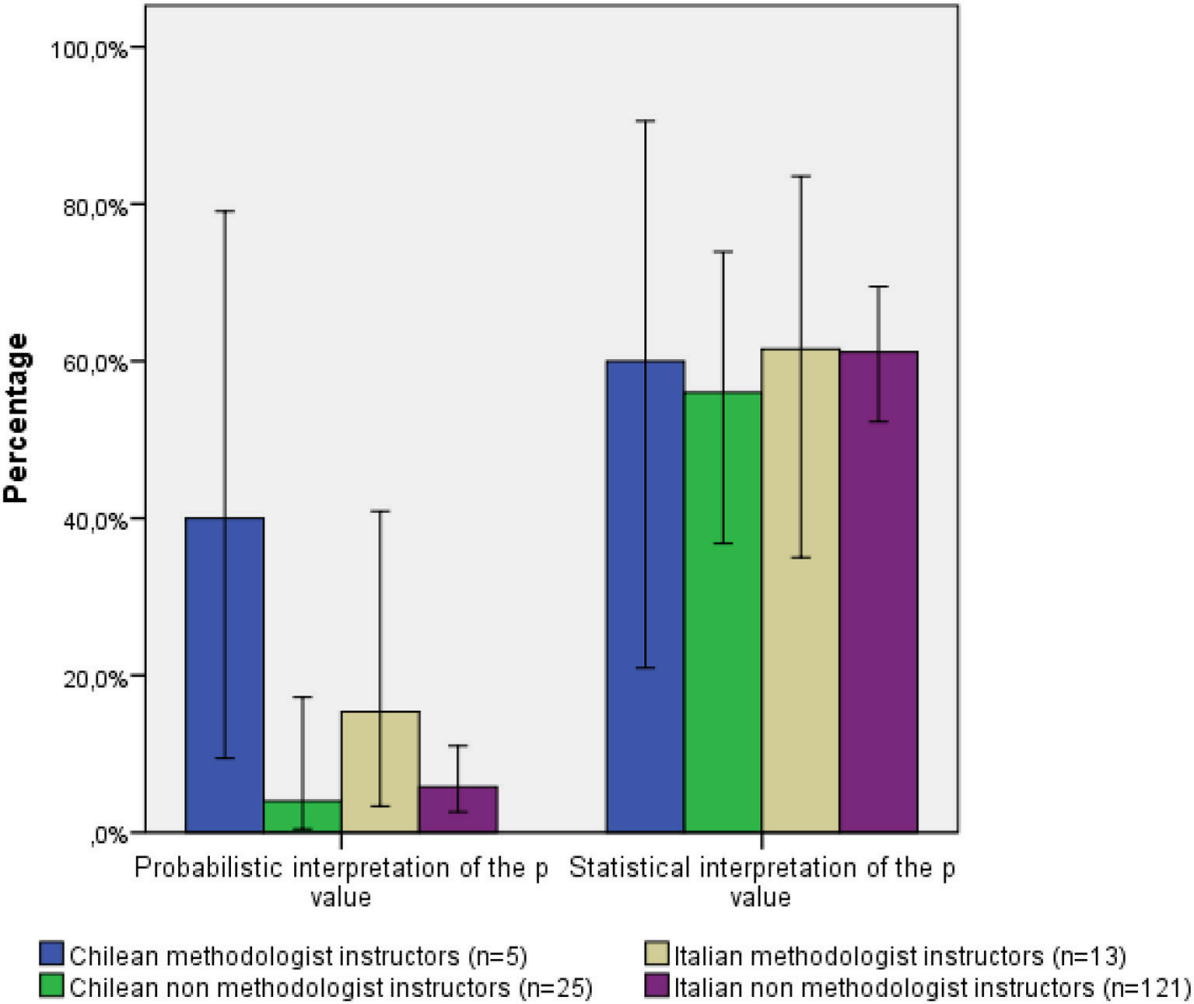
**Percentage of correct interpretation and statistical decision adopted broken down by knowledge area and nationality**.

Finally, Figure 2 shows the percentage of participants that endorsed each of the two correct statements. It can be noted that the majority of academic psychologists, including participants from Methodology area, had problems with the probabilistic interpretation of the *p*-value, unlike in the study of Badenes-Ribera et al. (2015a) where the Methodology instructors showed more problems with the interpretation of *p*-value in terms of the statistical conclusions (results not shown).

In addition, in all cases the interpretation of *p*-values improved when performed in terms of the statistical conclusions, compared to their probabilistic interpretation.

## Conclusion and discussion

Our work is a replication of the study by Badenes-Ribera et al. (2015a) which modified aspects of the original research design. In particular, we changed the answer scale format of the instrument for identifying misconceptions of *p*-values and the geographical areas of the participants. The latter aspect helps generalize the findings of the previous study to other countries.

Firstly, it is noteworthy that our answer scale format obtained a lower rate of misinterpretation of *p*-values than the original. Nevertheless, the difference between the studies might also be caused by a difference between countries.

In addition, our results indicate that the comprehension and correct application of many statistical concepts continue to be problematic among Chilean and Italian academic psychologists.

As in the original research, the “inverse probability fallacy” is the most frequently observed misinterpretation, followed by the “replication fallacy” in both samples. This means that some Chilean and Italian participants confuse the probability of obtaining a result or a more extreme result if the null hypothesis is true (Pr(Data|H_0_) with the probability that the null hypothesis is true given some data (Pr(H_0_|Data). In addition, not rejecting the null hypothesis does not imply its truthfulness. Thus, it should never be stated that the null hypothesis is “accepted” when the *p*-value is greater than alpha; the null hypothesis is either “rejected” or “not rejected” (Badenes-Ribera et al., 2015a).

In summary, as Verdam et al. (2014) point out, the *p*-value is not the probability of the null hypothesis; rejecting the null hypothesis does not prove that the alternative hypothesis is true; not rejecting the null hypothesis does not prove that the alternative hypothesis is false; and the *p*-value does not give any indication of the effect size. Furthermore, the *p*-value does not indicate replicability of results. Therefore, NHST only tells us about the probability of obtaining data which are equally or more discrepant than those obtained in the event that H_0_ is true (Cohen, 1994; Nickerson, 2000; Balluerka et al., 2009; Kline, 2013).

On the other hand, the results also indicate that academic psychologists from the area of Methodology are not immune to erroneous interpretations, and this can hinder the statistical training of students and facilitate the transmission of these false beliefs, as well as their perpetuation (Kirk, 2001; Haller and Krauss, 2002; Kline, 2013; Krishnan and Idris, 2014). This data is consistent with previous studies (Haller and Krauss, 2002; Lecoutre et al., 2003; Monterde-i-Bort et al., 2010) and with the original research from Spain (Badenes-Ribera et al., 2015a). Nevertheless, we have not found differences between academic psychologists from the area of Methodology and those from the other areas in the correct evaluation of *p*-values. It should however be borne in mind that lack of statistically significant differences does not imply evidence of equivalence. Furthermore, sample sizes in some sub-groups are very small (e.g., *n* = 5), yielding confidence intervals that are quite large. Thus, considerable overlaps of CIs for percentages are unsurprising (and not very informative). The interpretation of *p*-values improved in all cases when performed in terms of the statistical conclusion, compared to the probabilistic interpretation alone. These results are different from those presented in the original study (Badenes-Ribera et al., 2015a), but again the difference between the studies might be due to a difference between countries. In Spain, for example, literature about misconceptions of *p*-values has been available for 15 years (Pascual et al., 2000; Monterde-i-Bort et al., 2006, 2010), and this research can possibly be known by Methodology instructors. Therefore, it is possible that Spanish Methodologists are more familiar with these probabilistic concepts than their Italian counterparts. In this sense, language barriers may have posed a problem, Nevertheless, all literature published in Spanish was readable by Chilean researchers as well. Thus, lack of available literature cannot explain potential differences found between Spanish and Chilean researchers. In this last case, it is possible that differences exist in academic training between Spanish and Chilean Methodologists.

On the other hand, it can be noted that if participants consider the statement about probabilistic interpretation of *p*-values unclear, that may explain, at least partially, why it was endorsed by fewer lecturers than the statement on statistical interpretation of the *p*-value, both in the original and current samples. Future research should expand on this question using other definitions of *p*-value, such as by adding further questions like “the probability of witnessing the observed result or a more extreme value if the null hypothesis is true.”

Finally, it is noteworthy that these misconceptions are interpretation problems originating from the researcher and they are not a problem of NHST itself. Behind these erroneous interpretations are some beliefs and attributions about the significance of the results. Therefore, it is necessary to improve the statistical education or training of researchers and the content of statistics textbooks in order to guarantee high quality training of future professionals (Haller and Krauss, 2002; Cumming, 2012; Kline, 2013).

Reporting the effect size and its confidence intervals (CIs) could help avoid these erroneous interpretations (Kirk, 1996; Wilkinson and The Task Force on Statistical, Inference, 1999; Gliner et al., 2001; Balluerka et al., 2009; Fidler and Loftus, 2009; American Psychological Association, 2010; Coulson et al., 2010; Monterde-i-Bort et al., 2010; Hoekstra et al., 2012). This statistical practice would enhance the body of scientific knowledge (Frias-Navarro, 2011; Lakens, 2013). However, CIs are not immune to incorrect interpretations either (Belia et al., 2005; Hoekstra et al., 2014; Miller and Ulrich, 2016).

On the other hand, the use of effect size statistic and its CIs facilitates the development of “meta-analytic thinking” among researchers. “Meta-analytic thinking” redirects the design, analysis and interpretation of the results toward the effect size and, in addition, contextualizes its value within a specific area of investigation of the findings (Coulson et al., 2010; Frias-Navarro, 2011; Cumming, 2012; Kline, 2013; Peng et al., 2013). This knowledge enriches the interpretation of the findings, as it is possible to contextualize the effect, rate the precision of its estimation, and aid in the interpretation of the clinical and practical significance of the data. Finally, the real focus for many applied studies is not only finding proof that the therapy worked, but also quantifying its effectiveness. Nevertheless, reporting the effect size and its confidence intervals continues to be uncommon (Frias-Navarro et al., 2012; Fritz et al., 2012; Peng et al., 2013). As several authors point out, the “effect size fallacy” and the “clinical or practical significance fallacy” could underlie deficiencies in scientific reports published in high-impact journals when reporting effect size statistics (Kirk, 2001; Kline, 2013; Badenes-Ribera et al., 2015a).

In conclusion, the present study provides more evidence of the need for better statistical education, given the problems related to adequately interpreting the results obtained with the null hypothesis significance procedure. As Falk and Greenbaum (1995) point out, “unless strong measures in teaching statistics are taken, the chances of overcoming this misconception appear low at present” (p. 93). The results of this study report the prevalence on different misconceptions about *p*-value among academic psychologists. This information is fundamental for approaching and planning statistical education strategies designed to intervene in incorrect interpretations. Future research in this field should be directed toward intervention measures against the fallacies or interpretation errors related to the *p*-value of probability.

The work carried out by academic psychologists is reflected in their publications, which directly affect the accumulation of knowledge that takes place in each of the knowledge areas of Psychology. Therefore, their vision and interpretation of the findings is a quality filter that cannot be subjected to erroneous beliefs or interpretations of the statistical procedure, as this represents a basic tool for obtaining scientific knowledge.

## Limitations

We acknowledge some limitations of this study that need to be mentioned. Firstly, the results must be qualified by the low response rate, because of the 2321 academic psychologists who were sent an e-mail with the link to access the survey, only 164 took part (7.07%). The low response rate could affect the representativity of the sample and, therefore, the generalizability of the results. Moreover, it is possible that the participants who responded to the survey had higher levels of statistical knowledge than those who did not respond, particularly in the Chilean sample. Should this be the case, the results might underestimate the extension of the misconceptions of *p*-values among academic psychologists from Chile and Italy. Furthermore, it must also be acknowledged that some participants do not use quantitative methods at all. These individuals may have been less likely to respond, as well.

Another limitation of our study is the response format. By not asking to explicitly classify statements as either true of false, it is not possible to differentiate omissions from items identified as false. A three-response format (True/False/Don't know) would have been far more informative since this would have also allowed to identify omissions as such.

Nevertheless, the results of the present study agree with the findings of previous studies in samples of academic psychologists (Oakes, 1986; Haller and Krauss, 2002; Monterde-i-Bort et al., 2010; Badenes-Ribera et al., 2015a), statistics professionals (Lecoutre et al., 2003) psychology university students (Falk and Greenbaum, 1995; Haller and Krauss, 2002; Badenes-Ribera et al., 2015b) and members of the American Educational Research Association (Mittag and Thompson, 2000). All of this leads us to indicate the need to adequately train Psychology professionals to produce valid scientific knowledge and improve the professional practice. Evidence-Based Practice requires professionals to critically evaluate the findings of psychological research and studies. To do so, training is necessary in statistical concepts, research design methodology, and results of statistical inference tests (Badenes-Ribera et al., 2015a). In addition, new programs and manuals of statistics that include alternatives to traditional statistical approaches are needed. Finally, statistical software programs should be updated. There are several websites that offer routines/programs for computing general or specific effect size estimators and their confidence intervals (Fritz et al., 2012; Peng et al., 2013).

## Author contributions

LB, DF, BI, AB, and CL conceptualized and wrote the paper.

### Conflict of interest statement

The authors declare that the research was conducted in the absence of any commercial or financial relationships that could be construed as a potential conflict of interest.
